# Progress in Microtopography Optimization of Polymers-Based Pressure/Strain Sensors

**DOI:** 10.3390/polym15030764

**Published:** 2023-02-02

**Authors:** Shouheng Sun, Zhenqin Wang, Yuting Wang

**Affiliations:** 1 School of Economics and Management, University of Science and Technology Beijing, Beijing 100083, China; 2 Department of Physics, School of Mathematics and Physics, University of Science and Technology Beijing, Beijing 100083, China

**Keywords:** polymer-based sensors, pressure/strain sensors, topography optimization, structural performance optimization, wearable electronics

## Abstract

Due to the wide application of wearable electronic devices in daily life, research into flexible electronics has become very attractive. Recently, various polymer-based sensors have emerged with great sensing performance and excellent extensibility. It is well known that different structural designs each confer their own unique, great impacts on the properties of materials. For polymer-based pressure/strain sensors, different structural designs determine different response-sensing mechanisms, thus showing their unique advantages and characteristics. This paper mainly focuses on polymer-based pressure-sensing materials applied in different microstructures and reviews their respective advantages. At the same time, polymer-based pressure sensors with different microstructures, including with respect to their working mechanisms, key parameters, and relevant operating ranges, are discussed in detail. According to the summary of its performance and mechanisms, different morphologies of microstructures can be designed for a sensor according to its performance characteristics and application scenario requirements, and the optimal structure can be adjusted by weighing and comparing sensor performances for the future. Finally, a conclusion and future perspectives are described.

## 1. Introduction

With the rapid development of modern medicine, technology, and equipment, various innovative flexible electronic products have appeared in fields such as health detection, sports data statistics, body state detection, and other applications [1,2]. As an indispensable component of flexible electronic products, pressure sensors play an important role in converting external pressure into electrical signals [3]. Recently, with the rapid development of flexible stretchable substrates, stretchable transparent electrodes, flexible sensing materials, and new processing technologies [4], it has become possible to fabricate ultra-soft pressure sensors with high sensitivity, incredible flexibility, a large stretching range, biocompatibility, and low cost, and they are now capable of being mass produced. Compared with conductive bulk materials such as traditional metals and metal oxides, which show poor mechanical flexibility and limited elasticity, polymers—as flexible sensor substrates—are considered to be the most promising materials in this field because of their outstanding usability, flexibility, high transparency, low weight, and non-toxic characteristics. Moreover, polymers can be easily prepared and patterned by one-step solution methods (such as spin coating, shear coating, drop coating, or dip coating) and patterning technologies (such as ink-jet printing, screen printing, and three-dimensional printing) [5]. In addition, there are various preparation methods whereby polymer films with high conductivity and mechanical flexibility can be obtained, which can be used as stretchable electrode or flexible sensing materials. They present broad application prospects with respect to wearable devices for health monitoring, such as pressure sensing, heartbeat detection, motion detection, plantar pressure, etc. On the basis of traditional medical and health care devices that provide snapshots of human physiological conditions, polymer-based pressure sensors can realize real-time dynamic biological signal monitoring and more comfortable wearing experiences, offering better advantages in terms of accuracy, safety, functionality, and comfort [6,7].

Many functional polymer-based materials have been comprehensively investigated in order to assemble flexible tension sensors, such as polydimethylsiloxane (PDMS) [7,8,9,10], polyvinylidene fluoride (PVDF) [11], polyethylene terephthalate (PET) [12], polyurethane (PU) [13], polyimide (PI) [14,15], and Ecoflex [16]. Due to their superior flexibility, transparency, conductivity, and biocompatibility, these materials have been used to assemble flexible pressure sensors as flexible abstract/dielectric layers, sensing components, and conductive electrodes. However, with the development of society, people are demanding increasingly higher requirements for the performance of flexible pressure sensors. To obtain excellent properties and diverse functions, researchers have used microstructural design, that is, introducing small-scale microstructures on the surface or inside materials for their optimization. The microstructural design of materials not only significantly improves the sensitivity, detection limit, response time, and duality of flexible pressure sensors, but also allows for different structural designs corresponding to different performance characteristics and advantages. For example, a pyramid-structured pressure sensor can highly enhance the contact area of the sensor in response to low pressure because of the large stress concentration on the top of the sensor, thus enabling ultra-high response sensitivity. In contrast, a micro dome-structured sensor exhibits a wide sensing range due to the increasing number of new contact points and the increasing contact area under pressure, thus demonstrating the important role of structural design in the fabrication of high-performance and multifunctional flexible polymer-based mechanical sensors. Therefore, different microstructures dominate the sensing mechanism in each polymer-based pressure sensor and affect its performance indicators in different aspects.

In this review, we summarized the latest progress regarding the influence of different surface microstructures on the disconnection–reconnection mechanisms and performance of polymer-based pressure sensors, provided a reference for the design of ideal sensors in the future, and proposed the developmental direction of high-performance flexible pressure sensors. Firstly, a brief introduction to the commonly used polymers is provided in Section 2. In Section 3, the effects of different microstructures on the conduction mechanisms and properties of polymer-based piezoelectric materials are reviewed, and their respective advantages are discussed. Section 4 compares polymer-based pressure sensors with different microstructures, including their working mechanisms, key parameters, and relevant operating ranges. Finally, the conclusions and future perspectives are described.

## 2. Polymers

Various highly elastic polymeric materials have been selected as flexible substrates/dielectric layers as well as sensing materials and electrodes to assemble flexible pressure sensors due to their excellent deformability and simple manufacturing processes, including polydimethylsiloxane (PDMS) [17], poly(vinylidene fluoride) (PVDF) [17], polyvinyl alcohol (PVA) [18], polyethylene (PEN) [19], polyethylene terephthalate (PET) [20], polymethyl methacrylate (PMMA) [21], polyimide (PI) [12], polyurethane (PU) [22], polycarbonate (PC) [23], Ecoflex [24], epoxy [25], etc. Other conductive polymers such as polypyrrole (PPy) [26], polyaniline (PANI) [27], and hybrid poly(3,4-ethylenedioxythiophene) (PEDOT) [28,29] have shown higher electrical conductivity and excellent mechanical flexibility as wearable pressure sensors [30].

### 2.1. PDMS

Plenty of commercialized polymers are used as stretchable substrates in next-generation wearable strain/pressure sensors. Another important consideration of flexible polymers is their appropriate activity, which can be used in sensing elements. As a functional element, a sensing element can convert an external mechanical stimulus into an electrical signal. Among them, PDMS is the most widely used material because of its excellent comprehensive performance, not only as a flexible substrate, but also as a pressure-sensing layer. So far, PDMS-based composite pressure/strain sensors have been manufactured by combining various elements, such as metal nanomaterials (e.g., metal particles [31] and metal nanowires/networks [32,33,34,35]), carbon materials (e.g., carbon nanotubes (CNT), graphene/graphene oxide (GO), and carbon black (CB)), and other hybrid micro/nano-composites [36,37,38,39,40,41,42,43].

Polydimethylsiloxane (PDMS), as a kind of silicone elastomer that has proven to be a promising substrate due to its great stretchability, high thermal stability, chemical stability, simple processing, low price, etc. [44,45,46]. Due to its thinness, breathability, and excellent biocompatibility, it can be directly attached to human skin without causing allergic reactions or used as a flexible tissue-engineering material implanted in the body [47,48,49]. The high transparency of PDMS makes it an ideal substrate material for invisible electronic devices [50], which can be used as flexible electronic screens [50,51], glass and glasses sensors [52], contact lens sensors [53,54], wearable military sensing devices [55,56], etc. In addition, UV irradiation can be used to pattern such materials to make them locally adhesive, thereby providing a method for fabricating PDMS-based composite materials and integrated circuit systems [57]. As a dielectric material, PDMS with different microstructures and types of functionalization can be obtained through etching and pattern preparation and used for pressure sensing and tension sensing [58,59]. Based on the advantages of the material itself and the above processing technologies, its applicability in the field of pressure sensing has been further expanded [4,17,60].

### 2.2. PVDF

Polyvinylidene fluoride (PVDF), as a typical piezoelectric polymer, offers a low dielectric constant, remarkable sensitivity, high deformability, excellent thermal stability, and high chemical resistance, and is considered to be the most promising dynamic tactile sensing component among prospective wearable electronic devices. In order to obtain high-performance nanostructured PVDF materials, researchers have used various preparation methods, such as chemical vapor deposition (CVD) [61], sol–gel processes [62], hydrothermal methods [63], electrospinning [64], etc.

PVDF is a semicrystalline homopolymer with five phases (α, β, γ, δ, and ε phases), and can be obtained under various fabrication conditions [65]. PVDFs of different phases show different electric dipole moments from 5 to 8 × 10^−30^ C m and exhibit electric polarization change of electric dipole moment at different degrees when applying an external mechanical force, thus showing piezoelectric characteristics [66]. The piezoelectric properties of PVDF mainly depend on the polar crystalline phase, especially the highest electric dipole moment, namely, the β Phase [67]. Therefore, in order to increase the β Phase content in materials, researchers have proposed a variety of methods, including thermal annealing [68,69,70], uniaxial stretching [71], the use of a high electrical field [72,73], electrospinning [74,75,76], surface charge approaches [77,78], curing processes [79], etc.

Moreover, β Phase crystallization can be enhanced by adding nano fillers such as carbon materials [38,80,81], metal nanoparticles/nanofibers, [72,82] semiconductive ceramic [83,84], and other polymers [85,86]. The nanofibers of PVDF copolymers and their nanocomposites have been successfully prepared into oriented PVDF nanofibers, which were used as functional layers of piezoelectric sensors and piezoresistive sensors with excellent performance.

Among the materials in this category, poly (vinylidene fluoride trifluoroethylene) (P(VDF-TrFE)) has attracted much attention because the additional phase of TrFE can significantly improve the formation of β phases in order to obtain high piezoelectricity [81,87]. P(VDF-TrFE) also shows ferroelectric characteristics and excellent piezoelectric effects as well as great chemical stability and biological compatibility [88,89]. Moreover, via a facile fabrication process, P(VDF-TrFE) materials can be used to produce functional flexible tactile sensors with light weight, high detection performance, and low cost. This material can obtain a better piezoelectric response than PVDF film and offers a large effective working area and highly tensile structure [90,91].

Via a more directional arrangement of P(VDF-TrFE) fibers, piezoelectric performance of 40 times higher than the sensitivity of PVDF-based film pressure sensors can be obtained [87,92].

## 3. Polymer-Based Sensors

Different microstructural designs provide different types of performance for a sensor. According to the performance characteristics and application scenario requirements, researchers can design microstructures of different morphologies for sensors and select the optimal structure by weighing and comparing the sensor performance in terms of sensitivity, response time, detection limit, liner sensing range, duality, etc.

### 3.1. Pyramid Microstructure

Pyramid structures have been employed in pressure sensors because of their unique triangular structure. This special microstructure is usually accompanied by high sensitivity under low pressure, which is imparted by the sharp tip [93,94,95,96,97].

**Figure 1:**
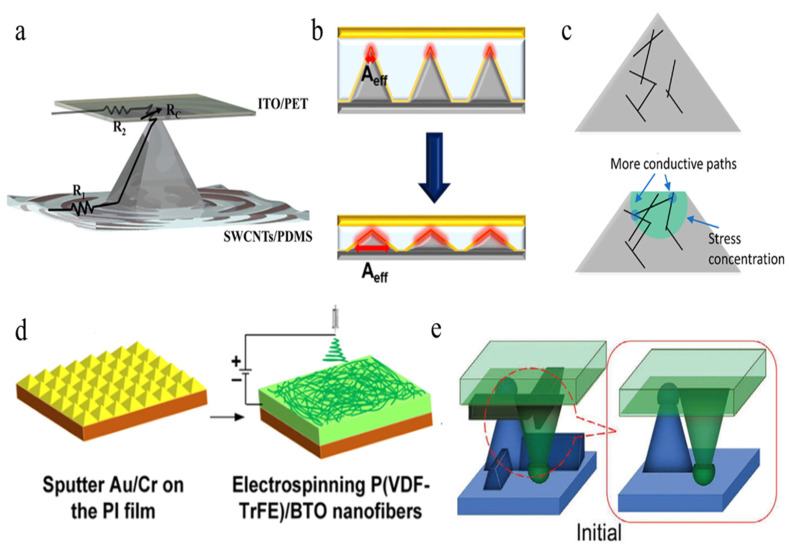
(**a**) Response mechanism of a SWCNT-coated polymer pyramidal pressure sensor [98]. (**b**) Mechanism of structural change of a transparent linear pressure sensor under pressure [99]. (**c**) Schematic of the conductive PDMS/CNT micropyramids, showing more conductive paths under stress [100]. (**d**) Schematic of fabrication process of electrospun fibers combined film with nanofiber mat [101]. (**e**) A pair of designed pyramids with dome-like tops touching the bottom film.

Figure 1a shows a mechanistic schematic diagram of the classic pyramidal microstructure pressure sensor [98]. When pressure is concentrated on the upper surface of the sensor, R1 and R2 will increase with the deformation of the soft surface substrate. As presented, the surface resistance (R1) and bottom resistance (R2) are several orders of magnitude smaller than the total resistance (Rc) of the sensor (greater than 1G ohm). Relative to the overall resistance of the sensor, R1 and R2 can be ignored. When the sensor surface is subjected to an external force, Rc will decrease with the increase in pressure due to the increasing contact area. Then, the small deformation of external load is converted into the change in the current signal under constant bias voltage. However, pyramidal microstructure sensors with high sensitivity show a very narrow pressure-sensing range.

To solve this problem, Park et al. [99] improved the pyramidal structure to increase the effective contact area, thus obtaining high linearity in a wide pressure range, as shown in Figure 1b. The capacitance change of the device is determined by the distance between the electrodes coated on the surface and the top of the pyramid and the contact area between the electrodes and the pyramid structure. Moreover, with the increase in pressure, the distance between the two electrodes and the contact area between the electrode and the pyramid structure increase linearly, which promotes the linear range of the capacitor. Moreover, Li etc. [100] modified the internal structure and further increased the number of conductive paths, resulting in a decrease in volume resistivity. Figure 1c shows that when stress is applied, the stress concentrated at the tip of the pyramid reduces the distance between the internal conductive networks of PDMS/CNT composites. As the pyramid’s height decreases, the contact area increases significantly, which leads to a decrease in contact resistance, thus increasing the device’s current. Subsequently, Zhang etc. [101] combined a nanofiber mat and a micropyramid array by electrospinning to increase their pressure sensitivity and greatly decrease their detection limit, as shown in Figure 1d. They developed a pressure sensor featuring micropyramid arrays (HD-μPA) and an active piezoelectric material composed of a poly(vinylidenefluoride-co-trifluoroethylene) [P(VDF-TrFE)]/barium titanate (BTO) nanofiber-based mat with silver nanowires (AgNWs) assembled as connected film electrodes. Consequently, the sensitivity was improved by about 1.7 times that of a flat substrate sensor because of the stress concentration on the top and the unbalance of the elastic modulus between the polymer-based nanofiber mat. At the same time, the pressure detection limit of the structural pressure sensor can be as low as 0.6 Pa, which shows great potential with respect to wearable applications.

Furthermore, Liu et al. [102] designed a flexible pressure sensor with a uniquely engineered pyramid wall grid microstructure (PWGM) on polydimethylsiloxane (PDMS) film, as shown in Figure 1e. The square pyramid with a dome-shaped head and a large top area and the reinforcing wall connecting all the pyramids endow the sensor with a series of resistance changes; consequently, first the pyramid and then the various walls contact and deform under vertical pressure. Meanwhile, the interconnection between the pyramid and the wall improves the crushing pressure of the PWGM PDMS film, and its new delamination deformation mechanism makes it highly sensitive and quickly responsive and endows it with long-term stability. The pressure sensor shows excellent sensitivity and mechanical durability of 383,665.9, 269,662.9, 48,689.1, and 1266.8 kPa^−1^ in the pressure ranges 0–1.6, 1.6–6, 6.1–11, and 11–56 kPa, respectively, thus showcasing its potential applications in wearable health-monitoring electronics. In conclusion, a single-row pyramid structure presents high sensitivity under micro pressure due to its special triangular pyramid structure.

According to Table 1, a pyramidal structure shows obvious deformation under low pressure; thus, pressure sensors of this structure always perform with ultra-high sensitivity under low pressure, which is suitable for monitoring weak signals such as respiration, pulse, etc.

### 3.2. Pillar Microstructure

In order to pursue both high sensitivity performance as well as a large and stable linear-sensing recognition area, researchers have designed pressure sensors with a pillar-shaped microstructure. By varying the pillar’s geometry, a pressure sensor with a desirable pressure-sensing region can be obtained. The micropillar array is applied to the pressure sensor with the help of the air gap between the pillar structure and the underlying substrate, and its response and relaxation time scales can reach milliseconds [103]. The current passing through the device will depend on the contact resistance between the covered micro-pillar and the substrate film and the resistance of the substrate film between two adjacent micropillars. By changing these two resistance parameters, pressure sensors with different sensitivities in different pressure areas can be designed [104].

**Figure 2:**
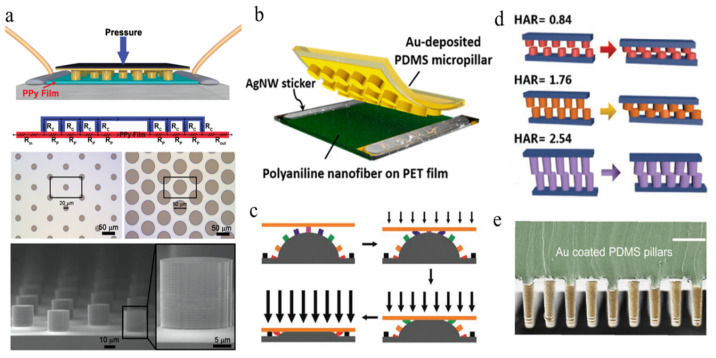
Schematic of micropillar array-based pressure sensor. (**a**) Detailed structure of pressure sensor based on micropillar array includes the PPy/PDMS substrate, a gold-covered micropillar array, and the optical images of photolithographically fabricated patterns [6]. (**b**) Primary design of the pressure sensor composed of PDMS micropillars and polyaniline nanofiber film [105]. (**c**) Schematic of in situ structural variation of hybrid structure under increased pressure load [106]. (**d**) Comparison of pillar-based pressure sensor with different ratio of height to diameter under pressure [107]. (**e**) SEM image of Au-coated PDMS micropillars. Scale bar: 50 μm [108].

Figure 2a shows a typical pressure sensor device composed of Au micropillar arrays and a deformable PPy/PDMS substrate film [6]. One micropillar-structured pressure sensor achieved high sensitivity in the low-pressure range by controlling the change in the contact resistance between the Au-coated micropillar surface and the yielding PPy film. Figure 2a shows a typical pressure sensor device composed of Au micropillar arrays and a deformable PPy/PDMS substrate film. The bottom of the figure shows a top view and a cross section of a regular and uniform pillar array. With a device composed of similar structures, Ha et al. [105] replaced the electrode with an improved Ag nanowire sticker to stabilize the electrical performance of pressure sensor on a deformable substrate under tension, as shown in Figure 2b. The polyaniline nanofibers are collected on a PET film as the bottom layer, and the gold-coated PDMS micropillars are used as the top layer.

More recently, different structural designs have been developed in order to improve sensitivity, such as that developed by Zhou et al. [106], which demonstrated a hybrid structure of interlocked micropillars and mesodomes (shown in Figure 2c). The increased number of micropillars brought about by a structural design featuring interlocked assembly not only increased the surface contact area but also caused larger pressure-induced deformation induced by the interlocking micropillars and mesodomes. Therefore, in terms of sensitivity and detectable pressure range, compared with the traditional structure based on single-fold domes, this hybrid structure shows excellent sensing performance.

To modify the micropillar structure, Hu et al. [107] discussed the influence of aspect ratios on micropillar structures by comparing the sensitivity of pressure sensors with three different height-to-diameter ratios, as shown in Figure 2d. It can be seen that the sensor with a greater height-to-diameter ratio shows higher sensitivity in the low-pressure range. Furthermore, Fang et al. [108] reported an Au-coated PDMS micropillar sensor with high-aspect-ratio microstructures, which can sense touch and detect weak physiological signals, even fingertip pulses. Figure 2e shows the skin–electrode mechanosensing structure of micropillars with a length (L) to radius (R) aspect ratio of 6. Unlike other sandwich structures, the skin–electrode mechanosensing structure consists of two electrodes, and the skin is designed to utilize ion transport in biological systems. Due to its ionic and electronic properties, the structure presents low noise but high signal strength when touched. Further mechanical analysis reveals that the instability of high-aspect ratio microstructures plays a critical role in sensing.

### 3.3. Microdome

Another form of microdome-shaped geometry has been developed to obtain a stable pressure sensor without experiencing fatigue during multiple cycles. Compared with pyramid and pillar structures where the pressure is concentrated at the tip, because a microdome’s geometry can expand the external tension more uniformly, this microstructure offers a stabler structure under pressure [109,110,111] and leads to a wider linear range and cyclic stability [112]. The linear range can be further widened by adjusting the microstructure of the sensing layer. Due to the special microdome shape of the elastomer, the contact area with the conductive electrode increases under external pressure, resulting in a significant reduction in the tunneling resistance or contact resistance. Although the sensors assembled with this structure show excellent sensing performance, they still suffer from problems such as the imbalance between high sensitivity and a wide linear range, which requires further optimization of the structure and composition [113,114].

**Figure 3:**
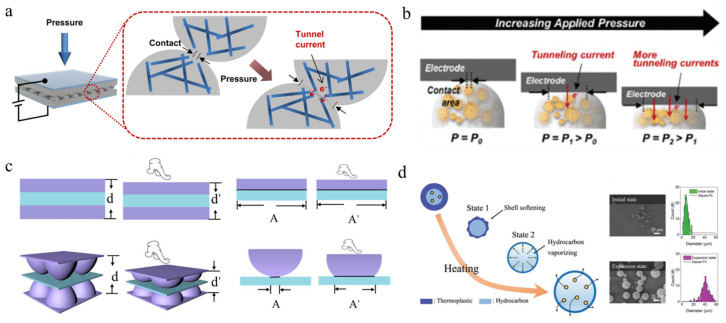
Schematic of microdome array-based pressure sensor. (**a**) Schematic of the working principle for interlocked microdome arrays with conductive nanofiber inside. Under pressure, the deformation of the micro dome increases the contact area and tunneling current of nanofibers [115]. (**b**) Schematic of microdomes composed of metal nanoparticles under pressure [116]. (**c**) Schematic diagram showing the difference in distance and contact area between film and microdome array under pressure [117]. (**d**) Thermal expansion of microdome with irregular structure under applied temperature [118].

To improve its sensitivity, Ko et al. used [115] a microdome structure design of interlocked microdome arrays, which display extreme resistance-switching behavior, including significant improvements in sensitivity and response/relaxation times. This massive improvement is achieved by the interlocking of the microdome arrays, which leads to an increase in the tunneling resistance at the contact point and the tunneling piezoresistance in the flexible film, as shown in Figure 3a. Later, Lee et al. [116] filled urchin-shaped metal nanoparticles into a polyurethane particle array to prepare a highly sensitive pressure sensor (71.37 kPa^−1^) with high optical transmittance (77.7% at 550 nm), as shown in Figure 3b. The excellent sensing performance of the transparent piezoresistive pressure sensor can be attributed to the effective quantum tunneling effect caused by the stress concentration at the small contact point and the deformation in the contact area.

Recently, Hu et al. [117] assembled a capacitive pressure sensor by adding a dielectric layer of polyvinylidene fluoride (PVDF) between two microdome-shaped conductive soft layers. For comparison, the internal stresses of the devices with flat and microdome-shaped conductive layers under pressure are shown in Figure 3c. As the contact area of the device with a flat conductive layer hardly changes under pressure, the relative capacitance of the device is only determined by the change in the dielectric layer’s thickness. In contrast, the microstructure in the devices with microdome-shaped conductive layers will deform significantly under pressure, leading to a change in the thickness and contact area of the dielectric layer. Hence, a noticeable improvement in sensitivity was observed in the microdome device. To modify the surface morphology of microdome arrays, Cho et al. [118] fabricated a 3D microstructure elastomer for flexible pressure sensors via the internal popping of microspheres. Each microsphere possesses a core–shell structure, consisting of thermoplastic resin as the shell and liquid hydrocarbon as the core. Figure 3d (left) shows the volume expansion process of the microspheres from state 1 to state 2 and, finally, the “expansion state” under a specific temperature. For a comparison of the volume change, Figure 3d (right) shows the SEM images and particle diameter distribution before and after the expansion.

### 3.4. Porous Sponge Microstructure

The structural characteristics of porous materials are similar to spongy and loose microstructures, which make them easier to deform under low pressure; thus, these materials exhibit a low detection limit and a larger deformation detection range. In addition, porous materials are very suitable for wearable flexible electronic devices due to their ultra-high illumination and good permeability. Researchers used an internal porous microstructure as a conductive or dielectric layer to fabricate lightweight pressure sensors. At the same time, the unique pressure detection function of porous microstructures in a large size range and in different planes enables them to be applied to detection in 3D space [119,120,121]. According to the requirements of different flexible wearable devices, such as light weight, low detection limit, and other characteristics, researchers have developed various porous microstructures with low density and high compressibility [122]. 

**Figure 4:**
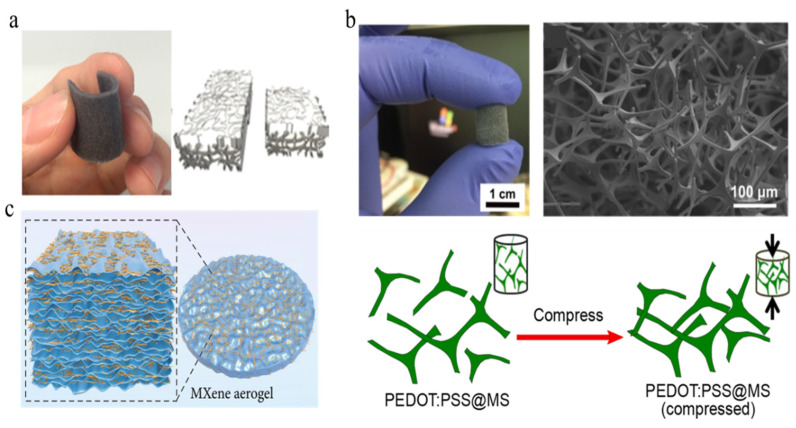
Schematic of pressure sensor based on porous composite material. (**a**) Photograph of a bendable porous composite sensor with GPN-PDMS and Schematic of the porous structure [124]. (**b**) Photograph of PEDOT:PSS-coated melamine sponge, SEM image of the porous sponge, and schematic of structural change under compression [129]. (**c**) Schematic of 3D porous sensor composed of MXene aerogel [137].

Due to the unique interconnective network of a porous sponge, electrons can move very quickly through the 3D network of the seamless interconnections of the conductive network, thus imparting very high electrical conductivity to the structure. It is very important to build a highly connected conductive filler network in the insulating matrix to improve the conductivity of the composite [123]. A typical fabrication process of a porous sponge-microstructured sensor is shown in Figure 4a [124]. This figure shows multilayer graphene grown on a nickel foam template by the chemical vapor deposition (CVD) method. Then, nickel foam coated with graphene sheets was immersed into prepared PDMS. The composite sample was subsequently cured in a hot plate at 100 °C, and the nickel skeleton was removed using hydrochloric acid. A composite of a graphene skeleton surrounded by PMDS was fabricated by removing the redundant PDMS. Moreover, the composite displayed good bending, torsional, and stretching properties. 

Recently, some conductive organic composite polymers have replaced the PDMS-based 3D frame structure, and the assembled sensors show good pressure-sensing abilities [125,126,127,128]. A conductive sponge copolymer composed of poly (3,4-ethylenedioxythiophene): poly (styrene sulfonate) (PEDOT: PSS) was constructed, and it presented a stable piezoresistive response under a compression strain of up to 80% [129]. Figure 4b shows a photograph and SEM image of the porous sponge sample as well as the structural changes during compression. Under compression force, the conduction mechanism is called the negative piezoresistive effect, which forms more conductive paths in the conductive elastomer composite or sponge, resulting in the lower resistance of the sensor. Due to the pores in the composite’s pressure and strain sensors, it can realize the strain-sensing characteristics of pressure and tension at the same time and shows a wide pressure-sensing range. The sponge reveals its robust porous morphology, high mechanical compressibility, high sensitivity, and stable compress−release cycles over 1000 cycles.

More recently, with the development of 1D nano/micro particles or 2D materials such as Fe_2_O_3_ particles, reduced graphene oxide, carbon nanofibers, MXene, etc. [130,131,132,133,134,135,136], Li et al. [137] successfully combined MXene with aerogel to increase its conductivity in order to assemble an interdigital sensor system, as shown in Figure 4c. The 3D porous MXene aerogel was composed of MXene nanosheets and cellulose nanofibers, which provide enough conductive channels with and without an external force. Finally, the 3D MXene aerogel was encapsulated in a PU layer to integrate a pressure-sensing device with self-healing properties. In addition, the MXene aerogel pressure sensor shows excellent response sensitivity of 306 kPa^−1^ with a wide pressure detection range from 2.3 Pa to 87.3 kPa and a fast response time of 35 ms. Moreover, it shows remarkable stability over 2000 cycles and confers self-healing characteristics to the system, constituting properties that can be applied in a variety of wearable sensor devices.

### 3.5. Bio-Inspired Microstructure

**Figure 5:**
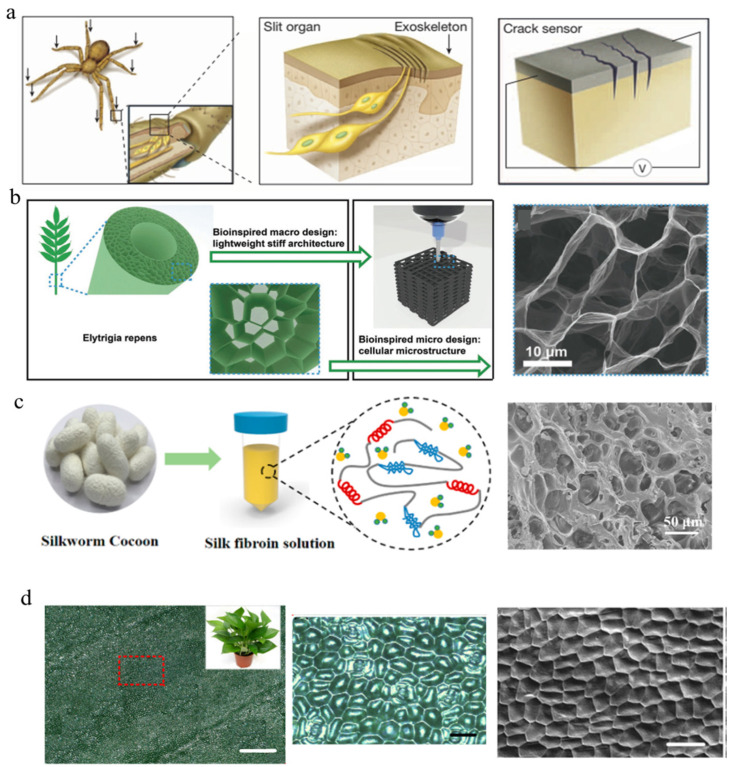
Schematic illustrations and images of bio-inspired pressure sensors. (**a**) Schematic of a spider’s sensory system and an ultra-mechanosensitive pressure sensor based on spider-inspired nanoscale crack junctions [31]. (**b**) Schematic of Elytrigia repens and 3D-printed hierarchical structure. SEM image of the hierarchical structure [140]. (**c**) Schematic illustration of the fabrication of the silk fibroin-based hydrogel. SEM image of interconnected porous architecture (right) [141]. (**d**) Optical images of an aureum leaf and SEM image of a replicated microstructured PDMS film. Scale bar: 1000 μm (**left**), 100 μm (**middle**), and 100 μm (**right**) [142].

Biomimetic materials are widely used in multi-functional flexible electronic materials [138,139]. In 2014, for the first time, Choi et al. [31] made use of nanoscale crack connections inspired by the geometry of a spider’s slit organs to prepare sensors with ultra-high sensitivity (gauge factor exceeds 2000) when detecting small vibrations (0–2% strain range). Figure 5a shows a schematic of the disconnection–reconnection mechanism of the sensor with ultra-high mechanical sensitivity and a zipper-like nanoscale crack connection under strain. Spiders use the slit organs on their leg joints to detect external vibrations and transmit signals to the nervous system. In this paper, the researchers mimicked the slit organ and obtained an ultra-sensitive strain sensor by preparing a crack-like Pt-coated viscoelastic polymer. When the coated Pt layer is as low as 20 nm thick, it forms cracks under mechanical bending. In addition, the detection sensitivity is greatly affected by the crack spacing (or density).

Later, Jiang et al. [140] reported a bionic hierarchical graphene material (BHGM) that exhibits ultra-high elasticity and stability when the compressive strain reaches 95%. The BHGM was constructed by imitating the structure of Elytrigia repens and is similar to an ice-crystal-induced cellular microstructure, as shown in Figure 5b (left). Elytrigia repens shows remarkable mechanical properties and light weight due to its gradually formed hierarchical structure and macro hollow structure and micro cellular structure. Researchers utilized an ink-based 3D printing strategy to prepare 3D multi-layer porously structured BHGMs (Figure 5b, right). The imitation of Elytrigia repens’s structure also grants the BHGM remarkably low weight and extremely high stiffness and elasticity.

Other structures such as silk fibroin-based hydrogels exhibit remarkable extensibility and compressibility, thus enabling them to be assembled into strain/pressure sensors with a wide range from 2% to 600% and good stability over multiple cycles [141]. Figure 5c shows the fabrication procedures of the composite hydrogel composed of silk fibroin, polyacrylamide, graphene oxide, and poly(3,4-ethylenedioxythiophene):poly(styrenesulfonate). The SEM images display the interconnected porous structure of the composite hydrogel, which leads to its excellent elastic and mechanical stability, thus allowing it to be manufactured into any shape.

Recently, researchers have used elastic PDMS to replicate the rough surface of Epipremnum aureum leaves and successfully prepared this bio-inspired microstructure [142,143]. Not only does this pressure sensor with a bio-inspired hybrid porous surface show an excellent sensitivity of 83.9 kPa^−1^ and excellent stability (>28,000 cycles), but it is also more attractive due to its ultra-low detection limit of less than 0.5 Pa, as shown Figure 5d. The optical images of a typical aureum leaf are shown in Figure 5d (left and middle) under scale bars of 1000 and 100 μm, respectively. In addition, the surface morphology of the replicated microstructured PDMS is shown on the right side of Figure 5d. The bio-inspired hybrid porous microstructure shows an increased contact area and reduced = Young’s modulus and introduces an additional level of pore resistance. Consequently, the pressure sensor exhibits high sensitivity and a low limit of detection. These aspects enable the designed sensor to detect finger pressure, sound vibrations, swallowing activity, and wrist pulse, and showcase its potential applications in artificial intelligence and flexible medical electronic applications. 

## 4. Performance Comparison

### 4.1. Comparison of Different Structures

**Figure 6:**
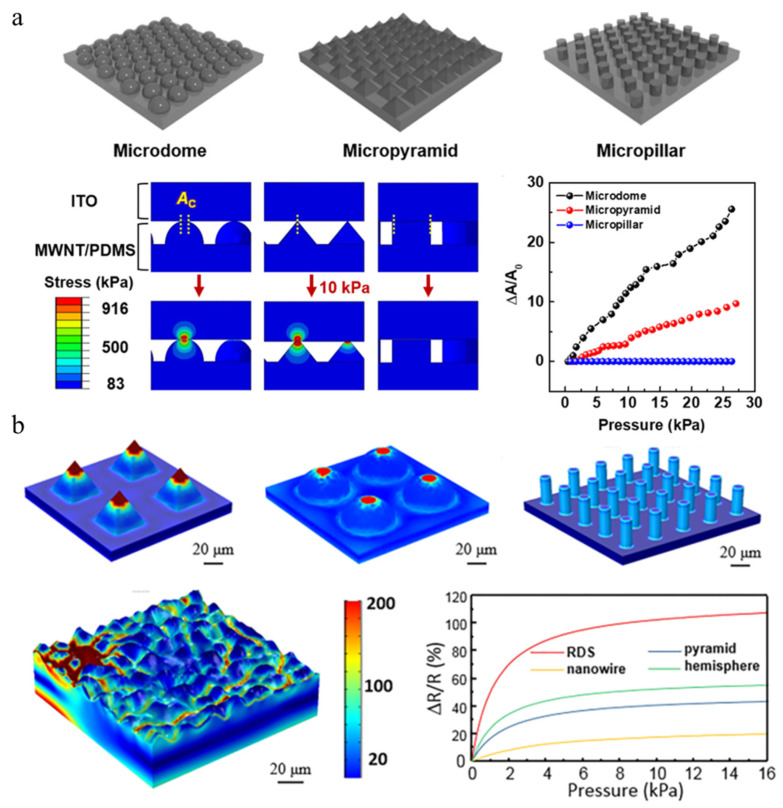
Comparison of the performance and mechanisms of pressure sensors with different microstructures. (**a**) Schematic illustration of differently microstructured arrays of pressure sensors. Calculation of localized stress distributions in response to pressure and relative contact-area changes in response to pressure determined by finite-element analysis (FEA) [144]. (**b**) The simulation results of pressure distribution and resistance variation versus applied pressure for different geometries under loading pressure [145].

Flexible pressure-sensing materials with various surface morphologies have different advantages and characteristics. To evaluate the effects of these different microstructures on the macrostructure of materials, researchers have compared several classical surface morphologies (pyramids, columns, and micro-bodies). Ko et al. [144] reported a sensitivity comparison of pressure-sensing materials with planar, microdome, micropillar, and micropyramid morphologies in different pressure detection ranges and simulated the contact area variation of different microstructures under normal pressure by a finite element method. The linear pressure sensitivity of devices with different non-planar surface morphologies shows much higher sensitivity (18.3, 12.6, and 5.3 kPa^−1^ for microdome, micropyramid, and micropillar structures, respectively) than that of a planar structure (0.5 kPa^−1^), especially in a pressure range of less than 1 kPa. In a planar structure, the main factor affecting the change in resistance is the inter-tube distance of the sandwich structure, while the contact area is basically unchanged. Compared with planar structures, other microstructures have smaller contact spots with the connected membrane electrodes, resulting in stress concentration, which increases the contact area and decreases the PDMS barrier thickness, ultimately increasing the tunneling current between the microstructure and the electrodes. In a medium pressure range, the pressure sensitivity decreases, but the trend of sensitivity for different microstructures remains unchanged (microdome > micropyramid > micropillar > planar), as shown in (Figure 6a). The finite element simulation is aimed at the stress distribution of the finite element simulation under the pressure of 10 kPa. It was found that the structural stress of the microdome and micropyramid is concentrated at the tip, resulting in a significant increase in sensitivity, as shown in Figure 6a.

A random distribution spinosum structure (RDS) shows superior sensing performance compared to that of a pyramid, hemisphere, or nanowire [145]. The simulation results in Figure 6b show the pressure distribution of pyramid, hemisphere, nanowire, and RDS structures under a 5 kPa load. For the pyramid and hemisphere structures, the stress is concentrated on the top of the tip area, and the nanowires show a uniform pressure distribution along the height direction. In contrast, the RDS shows a more uniform pressure distribution than that of the three other morphologies. The calculation shows that the stress in the RDS nanostructure concentrates at the contact tip and can be transferred to contiguous peak roots under the applied pressure, which indicates that the deformation in the low-pressure range is quite small. Therefore, surface treatment can confer great yield strength and a large linear range of strain and stress in the microstructure. Finally, the researchers concluded that the modified spinosum microstructure can achieve high sensitivity, while the randomly distributed spinosum morphology can achieve a large linear range of sensing performance.

### 4.2. Performances

The performance of polymer-based pressure/strain sensors is generally characterized by the following diagnostic parameters: sensitivity, response time, detection limit value, linearity range, and durability. This paper summarizes and compares the characteristic parameters of polymer-based pressure/strain sensors assembled with different microstructures, thereby providing a reference for strain/pressure sensor design [146,147].

First, sensitivity is considered to be the most important performance index of a pressure sensor, and it is defined as the slope of the pressure response curve, representing the ability of a device to convert pressure into an electrical signal. For piezoresistive and capacitive sensors, the pressure sensitivity is normally defined as S = δ(ΔX/X0)/δp, where ΔX is the variation in the signals; X0 denotes the initial signals, which can be resistance or capacitance; and p denotes the applied external pressure [148,149]. 

Next, the linear sensing range is a performance parameter used to evaluate the sensing stability range of a pressure sensor [150]. Generally, sensing materials with high sensitivity show great structural deformation or large changes in the conductive path at low pressure, which means that nonlinear resistance or capacitance changes will occur at higher pressure. Therefore, the preparation of pressure-sensing materials with both high sensitivity and a wide linear range has always been the goal of researchers. Recently, biomimetic pressure-sensing materials have made breakthroughs concerning the provision of high sensitivity and a large linear range at the same time [145,151,152]. There are other methods of determining sensitivity, such as using peak charge output to detect small vibrations [101].

Other important index parameters are the response time and relaxation time, which reflect the time resolution of the pressure sensor. Response time is the time required for the output signal to reach 90% of the maximum stable output value. Time resolution plays an important role in biomedical and biological detection [83,149]. Another method used to characterize response time is to count the frequency response, and the frequency signal can be easily converted into a pulse signal with any desired amplitude through data processing or additional circuits (such as an edge detector) [100]. High time resolution means faster and more accurate dynamic detection [37,43].

Finally, durability, as an important parameter of a pressure sensor in long-term practical use, is an important indicator of whether a sensing material can be commercialized for mass production and practical application. Researchers have greatly improved durability by mixing nano materials into sensing polymers [153].

After a long period of development, the reports based on polymer-based pressure sensors found that individual performance can be close to practical applications. However, it is difficult to achieve an overall balance for multiple functions and types of performance; thus, it is necessary to balance the sensitivity, linearity, and working range in the actual selection of the materials. Different microstructures can be designed according to the performance characteristics of the sensor and the requirements of the application scenario, thereby allowing for the best structure to be selected. To facilitate the comparison of the sensors developed in research, Table 1 lists the structural design and main evaluation parameters of representative polymer-based pressure sensors with different microstructures reported in recent years.

**Table 1:** Summary of main evaluation parameters of polymer-based flexible pressure sensors with different microstructures.

Polymers	Additive Agent	Technology	Micro Structure	Sensitivity	Pressure Detection Limit	Response Time	Linearity Range	Detection Range	Durability	Ref.
PDMS	–	Photolithography	Pyramid	~0.2 kPa^−1^	100 Pa	–	0–10 kPa	0–22 kPa	–	[93]
PDMS	Au particles	Plasma etching	Pyramid	383,665.9 kPa^−1^	0.25 Pa	75 ms	0–1.6 kPa; 1.6–6 kPa; 6.1–11 kPa	<60 kPa	1000 cycles	[102]
PDMS	CNT	Drop casting	Pyramid	0.34 kPa^−1^	–	10 to 120 Hz	10−60 kPa	0−120 kPa	Over 1000 cycles	[100]
PDMS	PEDOT:PSS layer	Photolithography, anisotropic etching, and CVD	Pyramid	0.034 kPa^−1^	14 Pa	210 ms	10–100 kPa	5–100 kPa	5500 cycles	[99]
PDMS	P(VDF-TrFE)/BTO Nanofiber	Ultraprecision microgroove fly cutting technology	Pyramid	Peak charge output of 47 pC	0.6 Pa	–	–	0.6–20 kPa	–	[101]
PVDF	Polyacrylamide/sodium-alginatehydrogel	Wet etching	Pyramid	12.4 kPa^−1^	–	–	0–50 kPa	–	100 cycles	[96]
PDMS	PPy film	Cutting and chemically depositing	Pillar	−1.8 kPa^−1^	2 Pa	decisecond	0–0.4 kPa	0–1 kPa	–	[6]
P(VDF-TrFE)	–	Spin coating and annealing	Pillar	458.2 mV/N	<2 Pa	2 Hz	0–4 N	–	36,000 cycles	[154]
PDMS	AgNWscoated	Spin coating and dip-coating	Pillar	128.29 kPa^−1^	2.5 Pa	0.2 ms	0–200 Pa	0–80 kPa	2000 cycles	[106]
PDMS	Au-coated	Photolithography and catalytic wet etching	Pillar	1.3–11.8 kPa^−1^	0.2 Pa	15 ms	<3 kPa	0–15 kPa	5000 cycles	[108]
PDMS	−	spin coating and oxygen plasma	Microdome	0.44% kPa^−1^	~55 Pa	288 ms	<11 kPa	<500 kPa	1000 cycles	[155]
PDMS	CNTs	Micromolding and sputter coating	Microdome	−15.1 kPa^−1^	∼0.2 Pa	∼0.04 s	<0.5 kPa	0–70 kPa	1000 cycles	[115]
PU	Metal nanoparticles	Soft molding	Microdome	71.37 kPa^−1^	4 Pa	30 ms	<100 Pa	∼1.5 k Pa	15 days under 70 °C and humidity 70%	[116]
PDMS	Microspheres	Spin coating and metal deposition	Microdome	−50.45 kPa^−1^	0.209 Pa	39 ms	0–50 Pa	0–400 Pa	4000 cycles	[118]
PU	Gold	Ion sputtering	Porous	59–122 Pa^−1^	0.568 Pa	9 ms	<10 kPa	∼14 kPa	1000 cycles	[126]
PDMS	Graphene	CVD and etching	Porous	0.09 kPa^−1^	–	100 ms	0–1000 kPa	0–3000 kPa	>10 cycles	[124]
PEDOT:PSS	Melamine	Cutting and dip coating	Porous	GF ≈ −2.32	1 Pa	3.5 s	1 Pa–1 kPa	0–35.0 kPa	1000 cycles	[129]
Aerogel	MXenes	Spraying and laser engraving	Porous	306 kPa^−1^	2.3 Pa	35 ms	0–2.5 kPa	2.3 Pa–87.3 kPa	>20,000 cycles	[137]
PDMS	Pt layer	Depositing	Bionics	2000	0–2%	–	∼5 Pa	0–2%	5000 cycles	[31]
PI	Metal electrodes	Spin coating and UV lithography	Bionics	22.4 kPa^−1^	7.3 ± 1.2 Pa	41 ms	<16 kPa	0 Pa–400 kPa	54,000 cycles	[156]
PEDOT:PSS; PAM/GO	Silkfibroin	Dissolving	Bionics	0.01374 kPa^−1^	∼2%; 0.5 kPa	170 ms	0−15.9	0.5−119.4 kPa	2000 cycles	[141]
PDMS	Ag thin-film	Molding and magnetron sputtering	Bionics	5.9 kPa^−1^	∼16 Pa	42 ms	0–15 kPa	0–30 kPa	2000 cycles	[143]

## 5. Conclusions and Perspectives

In conclusion, polymer-based pressure/strain sensors with different microstructures show various respective performance characteristics. PDMS and PVDF are the two most promising and common polymers used in this regard. PDMS has been applied to many soft tensile sensors due to its excellent dielectric constant and simple preparation process of various structural designs. However, PDMS-based pressure-sensing materials have the disadvantages of poor stability due to structural instability in the long-term tensile process cycles, the structural phase transition caused by temperature difference, and the brittle phase transition that occurs in a dry environment. In the future, an increasing amount of research will be needed regarding multiple cycle stability, reusability, and corrosion resistance under different environmental conditions. In addition, PVDF has been widely discussed and studied in recent years due to its special piezoelectric characteristics, which make it applicable to the preparation of self-powered wearable pressure sensors. However, the conversion efficiency of its piezoelectric performance and the stability of its signal transmission are far from those required in practical applications, thus necessitating further experiments on its structural optimization.

Among the polymer sensors with various structures, the pyramid structure shows high sensitivity under low pressure, while the pillar microstructure exhibits a larger and more stable linear sensing recognition area. Compared with the pyramid and pillar structures, microdome-structured sensors show better durability and a lack of fatigue over multiple cycles. Moreover, porous materials show a lower detection limit and a larger deformation detection range. The unique pressure detection function of porous microstructures across large size ranges and different planes enables their application to detection in three-dimensional space. Finally, as widely used, multi-functional, flexible electronic materials, bionic materials also show a variety of microstructures, including nanoscale cracks, hierarchical structures, porous structures, and sphenoid surfaces. Different structures show different performance characteristics. For example, nanoscale crack structures show ultra-high sensitivity with respect to detecting small vibrations along with high elasticity and stability. The compression strain range of hierarchical structures can reach 95%, while porous structures and sphenoid surfaces display a wide range and ultra-low operating voltage.

For the future development of ideal flexible pressure sensors, such sensors need to have both ultra-high response sensitivity and an ultra-large sensing range, but these two parameters are often contradictory. The development of these two parameters of the sensing material such that they are reasonable and balanced constitutes an important problem that researchers will need to solve. In this paper, the effects of various microstructures on sensing performance have been summarized. Different morphologies of microstructures can be designed for a sensor according to the desired performance characteristics and application scenario requirements, and the optimal structure can be adjusted by weighing and comparing sensor performance (e.g., sensitivity, response time, detection limitation value, linear range, and duality).

In addition, the development of a flexible bifunctional or multifunctional sensor is an important topic for future research. Multi-functionality can be achieved by designing and preparing multi-functional materials [157,158] or integrating materials with various sensing abilities [159,160]. Most of the reported sensor devices are not an integrated unit but have a simple layout with two separate sensing elements lying in parallel on the same side of the substrate. Future efforts will be directed towards determining a method with which to integrate the sensing elements into multi-functional devices and thus realize applications in flexible electronic systems. Lastly, in addition to the above sensing performance-related problems, the comfort of flexible sensors when worn for a long time in practical applications has also attracted an increasing amount of attention with respect to aspects such as breathability, biocompatibility, waterproofing, the prevention of sensory interference, etc. [18,161,162]. Therefore, the development of new types of ultra-thin flexible sensors with excellent breathability and anti-perspiratory properties in order to achieve comfortable long-term wear is another research direction.

## Data Availability

All data generated or analyzed during this study are included in this published article.
